# Synthesis, Characterization, and Photovoltaic Properties of Soluble TiOPc Derivatives

**DOI:** 10.3390/ijms9122745

**Published:** 2008-12-19

**Authors:** Young-Keun Kim, Hyo-Jin Kang, Young-Wook Jang, Su-Bin Lee, Seung-Min Lee, Ki-Suck Jung, Jin-Kook Lee, Mi-Ra Kim

**Affiliations:** 1 Department of Polymer Science and Engineering, Pusan National University, Busan, 609-735, South Korea; 2 Polycen Co., Ltd., Busan, 608-739, South Korea; 3 Daehan Solvay Special Chemicals Co., Ltd., Ulsan, South Korea

**Keywords:** Titanyl phthalocyanine (TiOPc), Dye-sensitized solar cell, Polymer electrolyte

## Abstract

We have synthesized soluble TiOPc derivatives containing alkoxy groups for use as additives in dye-sensitized solar cells (DSSCs). The DSSC devices containing these TiOPc derivatives exhibited short-circuit current densities of 8.49~10.04 mA/cm^2^ and power conversion efficiencies of 2.73~3.62 % under AM 1.5 illumination and 100 mW/cm^2^ irradiation.

## 1. Introduction

Dye-sensitized solar cells (DSSCs) constructed using dye molecules, nanocrystalline metal oxides and organic liquid electrolytes were originally developed by the research group of Gratzel *et al*. [1–6]. DSSCs have attracted much attention around the world, due to their high power conversion efficiency, easy fabrication, and low production cost. However, DSSCs have not been used in practical applications because many difficulties regarding their use of a liquid electrolyte remain unresolved, such as solvent evaporation, leakage and deterioration, which cause sealing problems and performance degradation. To solve these problems, solid-state electrolytes have been developed to replace these liquid electrolytes. Solid-state DSSCs do not need hermetic sealing, but their power conversion efficiency is lower than that of DSSCs with conventional organic liquid electrolytes. To improve their power conversion efficiency, attempts have been made to add various materials to the electrolyte [7–12]. Solid-state or quasi-solid-state electrolytes, such as hole-conducting molecular solids [13] and polymers [14], molten salts [15] and ionic liquids [16–17], have been investigated and used in place of the volatile organic solvent in DSSCs. In light of their unusual electrochemical and electronic properties, phthalocyanines (Pcs) are potentially useful additives. Pcs have attracted the attention of many researchers during the twentieth century and are still being actively studied in this century [18–19]. Many potential applications are expected for phthalocyanines (Pcs), which have high thermal and chemical stability, for instance as solar cell functional materials, gas sensors, and photodynamic therapy agents. The results concerning DSSCs using TiOPc as a co-adsorbent have been previously reported [20]. However, many of the applications of Pcs have been limited by their lack of solubility in organic solvents and water. Over the past few decades, a large variety of substituted Pc derivatives have been synthesized in order to improve their solubility [21, 22].

In this study, we synthesized titanyl phthalocyanine (TiOPc) derivatives containing an alkoxy chain group with good solubility. The molecular structures of the synthesized TiOPc derivatives were characterized by Fourier Transform Infrared Spectrophotometry (FT-IR), Matrix Assisted Laser Desorption Ionization Time-of-Flight (MALDI-TOF) mass spectroscopy, X-ray Diffraction (XRD), and Transmission Electron Microscopy (TEM). The thermal properties of the TiOPc derivatives were analyzed by thermal gravimetric analysis (TGA) and differential scanning calorimetry (DSC) under a nitrogen atmosphere. The optical properties of the TiOPc derivatives were monitored by UV-vis spectrophotometry and fluorescence spectrophotometry.

We also studied the photovoltaic properties of the TiOPc derivatives. We prepared quasi-solid state dye-sensitized solar cell (DSSC) devices with TiOPc derivatives. These DSSC devices were fabricated using ruthenium (II) complex dye (N3 dye) as a photosensitizer and the TiOPc derivatives as additives in the electrolytes.

## 2. Results and Discussion

### *2.1 Synthesis and characterization of TiOPc derivatives* (**3a** and **3b**)

**Scheme 1.**
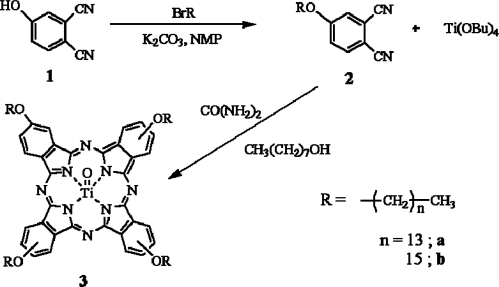
The synthetic route to titanyl phthalocyanine (TiOPc) derivatives.

The TiOPc derivatives, 2,9,16,23-tridecyloxyphthalocyaninato oxotitanium(IV) (**3a)** and 2,9,16,23-pentadecyloxyphthalocyaninato oxotitanium(IV) (**3b)** were prepared using the two-step synthesis described in Ref. [23]. The synthetic route of TiOPc derivatives is shown in Scheme 1. Their chemical structures were characterized by FT-IR. The characteristic alkyl group stretch at 2850–2950 cm^−1^ and ether stretch at 1250 cm^−1^ of 4-tridecyloxyphthalonitrile (**2a**) / 4-pentadecyloxyphthalonitrile (**2b**) appear upon the formation of the alkoxy group in alkoxyphthalonitriles. The characteristic nitrile (C≡N) stretch at 2232 cm^−1^ of **2** disappears upon the formation of the TiOPc. The split ether stretching frequencies are prominent for both the phthalonitriles and the phthalocyanines in the 1100–1264 cm^−1^ range.

### 2.2. Solubility of **3a** and **3b**

The solubilities of **3a** and **3b** were examined at a ratio of compound to solvent of 100 mg/mL. Table 1 shows the solubilities of TiOPc, **3a**, and **3b** in a variety of common solvents. TiOPc was insoluble in almost all organic solvents. Compared with TiOPc, **3a** and **3b** exhibited increased solubility in various solvents, such as chloroform, chlorobenzene, and toluene, but not in methanol and acetone. In many applications, the solubility of materials is a very important problem. Therefore, the solubility of **3a** and **3b** in organic solvents makes them promising materials.

### 2.3. Optical properties of **3a** and **3b**

**Figure 1.**
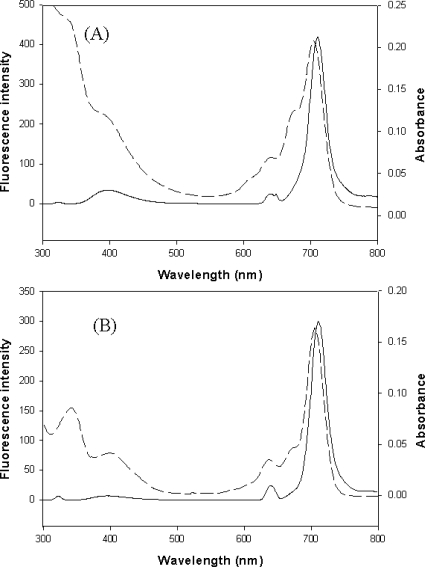
Fluorescence (solid line) and absorption (dashed line) spectra of (A) **3a** and (B) **3b**.

The absorption and fluorescence spectra of **3a** and **3b** in chloroform are shown in Figure 1. The absorption spectra of **3a** and **3b** showed broad bands in the region around 340 nm and sharp peaks at 704 nm and 705 nm, respectively. Weak Q-band region peaks contributed by the aggregations appear in the 620–680 nm region. The spectra show the typical Soret and Q-bands, which are characteristic of phthalocyanines. Upon excitation at 640 nm. Compounds **3a** and **3b** showed fluorescence emissions at 709 and 711 nm, respectively.

### 2.4. XRD

The XRD patterns of **3a** and **3b** measured in the range of 2 theta of 10–50 degrees (Table 2) show identical features with relatively poor crystallinity. Although the observed patterns resemble qualitatively that of the corresponding unsubstituted TiOPc, the peaks are found to be broadened with diffused intensity. This reveals that **3a** and **3b** were less crystalline than the unsubstituted TiOPc [24]. This may be due to the presence of the bulky substituent alkoxy chain, and seems to play a dominant role in the stacking of the metal phthalocyanine derivatives. The X-ray diffraction patterns were only used to explain the degree of crystallinity, which is qualitative. The effect of the alkoxy chain substitution can be clearly identified from the first d values of all of the complexes.

### 2.5. TEM studies

**Figure 2.**
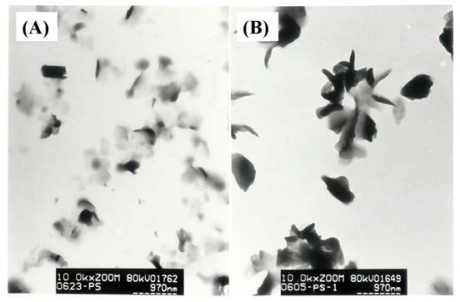
TEM images of **3a** (A) and **3b** (B).

The size and morphology of the synthesized compounds were analyzed by TEM measurements. The TEM images of **3a** and **3b** are shown in Figure 2. The TEM micrography revealed that **3a** and **3b** consisted of irregular spherical nanoparticles with diameters ranging from 450 nm to 600 nm and that the particles were agglomerated.

### 2.6. AFM studies

**Figure 3.**
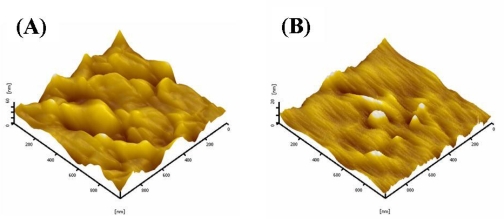
AFM images of **3a** (A) and **3b** (B); image sizes are 3 μm × 3 μm.

The surface morphology of **3a** and **3b** films was measured by atomic force microscopy (AFM). All of the films were prepared by the spin-coating method from a chloroform solution. The difference in the root mean square (RMS) surface roughness between the two films was not very large, as depicted in Figure 3. The RMS roughnesses of **3a** and **3b** were 2.22 nm and 10.59 nm, respectively.

### 2.7. TGA and DSC studies

**Figure 4.**
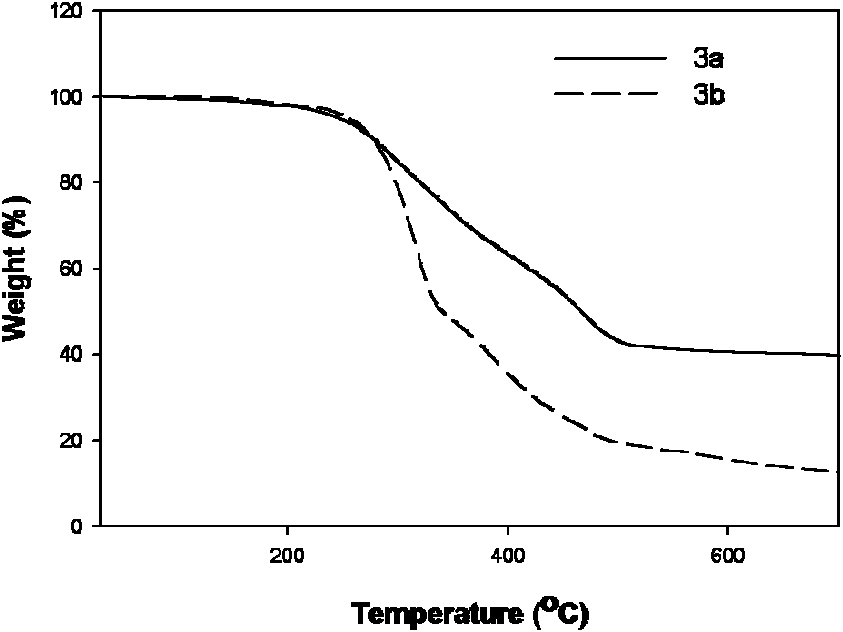
TGA curves of **3a** and **3b**.

**Figure 5.**
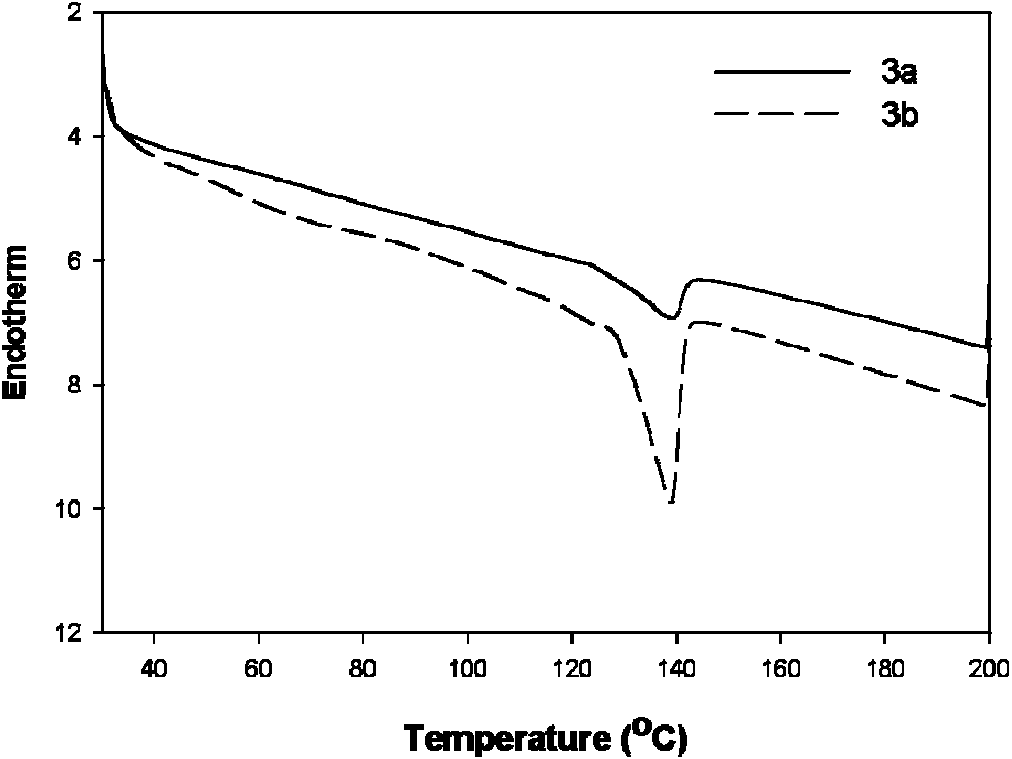
DSC curves of **3a** and **3b**.

The TGA curves of **3a** and **3b** are illustrated in Figure 4. The typical TGA curve obtained with a heating rate of 10 °C/min demonstrated high thermal stability up to 245 °C. The initial decomposition temperatures (Tds) of **3a** and **3b** were observed to be 213.65 °C and 256.11 °C, respectively, which are less than that of TiOPc. This may be due to the substituted alkoxy chains of **3a** and **3b**. Figure 5 shows the DSC curves of 3a and 3b.

### 2.8. Photovoltaic performances of DSSC devices

**Figure 6.**
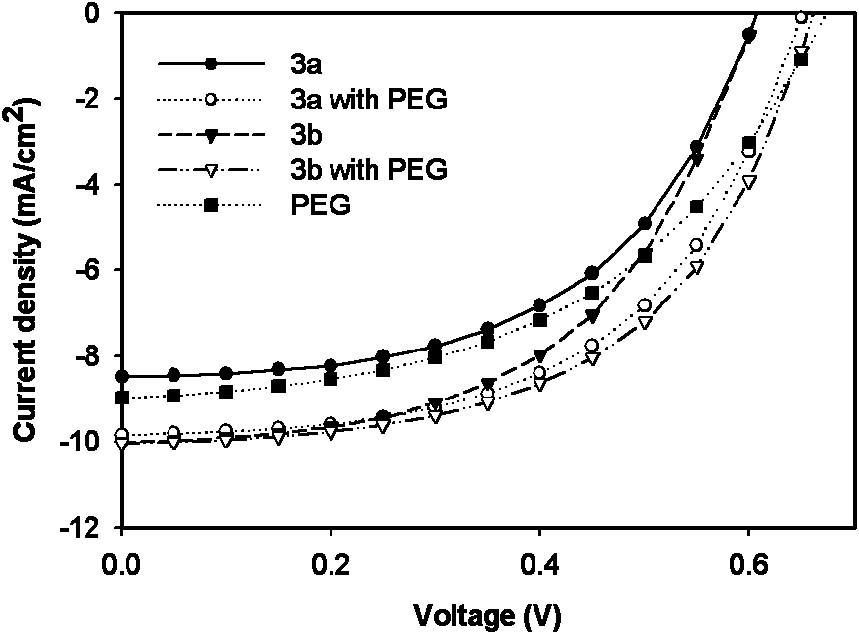
I–V curves of FTO/TiO_2_/Dye/Electrolyte/Pt devices using **3a**, **3a** with PEG, **3b**, **3b** with PEG, and PEG under AM 1.5 illumination; light intensity: 100 mW/cm^2^; active area: 0.25 cm^2^; with mask.

The photovoltaic measurements were performed using a solar simulator under AM 1.5 illuminated conditions, and the active area of the DSSC devices was 0.25 cm^2^. The power conversion efficiency (η) of a solar cell given by:

(1) $$\begin{array}{c} \eta ={\text{P}}_{\text{out}}/{\text{P}}_{\text{in}}=({\text{J}}_{\text{sc}}\times {\text{V}}_{\text{oc}}\times \text{FF}){\text{P}}_{\text{in}}\\ \text{with}\;\text{FF}=({\text{I}}_{\max}\times {\text{V}}_{\max})/({\text{J}}_{\text{sc}}\times {\text{V}}_{\text{oc}})={\text{P}}_{\max}/({\text{J}}_{\text{sc}}\times {\text{V}}_{\text{oc}}) \end{array}$$

where P_out_ is the output electrical power of the device under illumination and P_in_ represents the intensity of the incident light (e.g., in W/m^2^ or mW/cm^2^). V_oc_ is the open-circuit voltage, J_sc_ is the short-circuit current density, and the fill factor (FF) is calculated from the values of V_oc_, J_sc_, and the maximum power point, P_max_. Figure 6 shows the I–V curves of the FTO/TiO_2_/Dye/Electrolyte/Pt device using **3a** or **3b** as an additive. The DSSC devices using the TiOPc derivatives showed different results according to the methyl chain length. The values of V_oc_, J_sc_, FF and the power conversion efficiency (η) are listed in Table 3. The J_sc_s of the devices using **3a**, **3a** with PEG, **3b**, **3b** with PEG, and PEG were 8.49, 9.84, 10.02, 10.04, and 8.98 mA/cm^2^, respectively. The power conversion efficiencies of the DSSC devices using **3a**, **3a** with PEG, **3b**, **3b** with PEG, and PEG was 2.73, 3.49, 3.19, 3.62, and 2.94 %, respectively. The DSSC devices using **3a** and **3b** with PEG showed higher photovoltaic performance than the devices using PEG without **3a** and **3b** in the same procedure. The J_sc_ values of the DSSC devices using **3a** and **3b** were increased, and this can attributed to improvements in their power conversion efficiency.

## 3. Experimental Section

### 3.1. Materials

4-Hydroxyphthalonitrile, 1-bromohexadecane, 1-bromotetradecane, 1-octanol, 1-methyl-2-pyrrolidinone (NMP), titanium(IV) butoxide [Ti(OBu)_4_], I_2_, tetrabutylammonium iodide (TBAI), ethylene carbonate (EC), and propylene carbonate (PC) were purchased from Sigma-Aldrich Co. Urea was purchased from Shinyo Pure Chemicals Co. All reagents were of analytical grade and used as received without further purification. TiO_2_ paste, viz. Ti-Nanoxide HT/SP (particle size: 9 nm, wt 20 %), cis-bis(isothiocyanato)bis(2,2′-bipyridyl-4,4′-dicarboxylato)-ruthenium(II) dye (N3 dye), F-doped SnO_2_ glass (FTO glass), Pt paste (Pt catalyst T/SP), and 1-propyl-3-methylimidazolium iodide (PMImI), were purchased from Solaronix CA.

### 3.2. Measurements

The FT-IR spectra (KBr pellets) were recorded on a Jasco FT/IR-460 Plus spectrometer. The ^1^H NMR spectra (300 MHz) were recorded in CDCl_3_ using a Varian Unity Plus 300 NMR spectrometer. The UV-vis absorption and fluorescence spectra of the TiOPc derivatives in chloroform solution were recorded on a UVIKON 860 spectrophotometer and Hitachi F-4500 fluorescence spectrophotometer, respectively. The TEM images were recorded on a Hitachi H-7500 Transmission Electron Microscope. Scanning Probe Microscopy was performed using a NITECH Model SPA-400. The TGA and DSC analyses were conducted on a TA instruments (TGA-Q 50 and TGA-Q 100) thermal analyser at a heating rate of 10 °C/min under flowing nitrogen (40 mL/min and 50 mL/min). The measurement of the I–V characteristics of the solar cells was carried out using a Solar Simulator (300 W simulator, models 81150) under simulated solar light with an ARC Lamp power supply (AM 1.5, 100 mW/cm^2^).

### 3.3. Synthesis of TiOPc derivatives **3a** and **3b**

In the first step, alkoxyphthalonitriles were formed from 4-hydroxyphthalonitrile and 1-bromo-hexadecane or 1-bromotetradecane. 4-Hydroxyphthalonitrile (0.72 g, 5 mmol) and dry potassium carbonate (5.52 g, 40 mmol) was stirred for 30 min under N_2_ gas in NMP, then a solution of 1-bromohexadecane or 1-bromotetradecane (10 mmol) in NMP was added and the mixture was stirred for 12 h at room temperature. The reaction mixture was filtered and purified by chromatography on a silica column with dichloromethane as the eluent. Yield: 92 % (2a) and 79 % (2b).

**2a**; FT-IR (KBr, cm^−1^) : 2917, 2852 (C-H str.), 2232 (C≡N), 1601, 1562 (Ar. C=C str.), 1475 (CH_2_ bend), 1429 (CH_3_ bend), 1308, 1251 (C-O); ^1^H-NMR (δ) 7.72, 7.25, 7.20 (Ar. C-H), 4.05 (-O-CH_2_-), 1.83 (-O-CH_2_-CH_2_-), 1.60 (-CH_2_-CH_3_), 1.46, 1.27 (-CH_2_-), 0.89 (-CH_3_); Anal calc. for C_22_H_32_N_2_O: C 77.60, H 9.47, N 8.23, O 4.70; found : C 76.30, H 12.20, N 8.17; MS: 340; **2b**; FT-IR (KBr, cm^−1^): 2917, 2851 (C-H str.), 2232 (C≡N), 1603, 1562 (Ar. C=C str.), 1475 (CH_2_ bend), 1431 (CH_3_ bend), 1308, 1252 (C-O); ^1^H-NMR (δ) 7.74, 7.29, 7.20 (Ar. C-H), 4.06 (-O-CH_2_-), 1.86 (-O-CH_2_-CH_2_-), 1.60 (-CH_2_-CH_3_), 1.46, 1.27 (-CH_2_-), 0.89 (-CH_3_); Anal calc. for C_24_H_36_N_2_O: C 78.21, H 9.85, N 7.60, O 4.34; found : C 79.22, H 13.31, N 8.02; MS: 368;

The second step was the base catalyzed cyclotetramerization of the phthalonitriles. A mixture of alkoxyphthalonitrile (4 mmol), Ti(OBu)_4_ (0.37 g, 1.1 mmol), urea (0.12 g, 2 mmol), and 1-octanol was heated at 150 °C under N_2_ for 24 h. After the addition of methanol to the reaction mixture followed by refluxing for 30 min, the resulting deep green blue crystals were collected by filtration, washed with methanol, and then dried in a vacuum oven at 100 °C. Yield: 24 % (**3a**) and 21 % (**3b**).

**3a**; FT-IR (KBr, cm^−1^) : 2920, 2850 (C-H str.), 1607, 1529 (Ar. C=C str.), 1529, 1468 (CH_2_ bend), 1383, 1344, 1282 (C-N), 1244, 1120 (C-O), 1074, 1016, 965, 749 (Ti-N); MS MALDI-TOF: 1364 (MH^+^), **3b**; FT-IR (KBr, cm^−1^): 2915, 2854 (C-H str.), 1752, 1607, 1531 (Ar. C=C str.), 1492, 1464 (CH_2_ bend), 1367, 1343, 1302 (C-N), 1237, 1120 (C-O), 1073, 1016, 964, 750 (Ti-N); MS MALDI-TOF: 1476 (MH^+^).

### 3.4. Fabrication of DSSC devices

**Figure 7.**
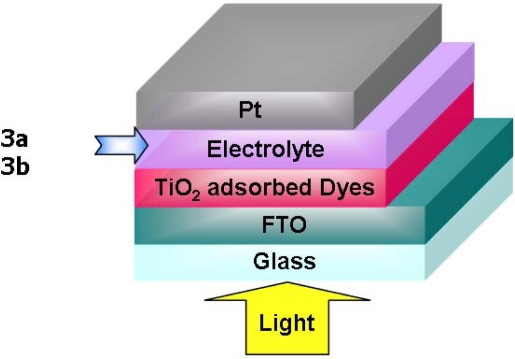
The structure of FTO/TiO_2_/N3 Dye/Electrolyte/Pt device using **3a** and **3b**.

We prepared DSSC devices using a quasi-solid state electrolyte containing **3a** or **3b** as an additive sandwiched with TiO_2_ adsorbed dyes and Pt-coated electrode as the two electrodes. The structure of the DSSC device is shown in Figure 7. The FTO/TiO_2_/Dye/Electrolyte/Pt device was fabricated using the following procedure; a volume of ca. 10 μL/cm^2^ of the transparent paste (Ti-Nanoxide HT) was spread on FTO glass by the doctor blade method. After heating the FTO glass covered with TiO_2_ nanoparticles successively at ca. 100 °C and ca. 450 °C for about 30 min each, the sintering process was completed and the TiO_2_ deposited electrode was cooled down from 100 °C to ca. 60 °C at a controlled cooling rate (5 °C/min) to avoid the cracking of the glass. A Pt counter electrode was fabricated by spreading on FTO glass using the doctor blade method. After heating the FTO glass spread Pt catalyst T/SP at 100 °C for 10 min, it was fired at 400 °C for 30 min. N3 dye was dissolved in absolute ethanol at a concentration of 20 mg per 100 mL of solution. Nanoporous TiO_2_ film was dipped in this solution at room temperature for 24 hours. Afterwards, the dye-sensitized TiO_2_ electrode was rinsed with absolute ethanol and dried in air. Without a sealant, the electrolyte solution was cast onto the TiO_2_ electrode impregnated with N3 dye and then dried at 55 °C for 2 hours. The electrolyte solution was composed of 24 mg of I_2_, 72 mg of TBAI, 80 mg of PMImI as an ionic liquid, 0.32 mL of EC/0.08 mL of PC (EC/PC=4/1 as volume ratio) and **3a** or **3b** in acetonitrile solution.

## 4. Conclusions

We have synthesized two kinds of TiOPc derivatives containing alkoxy groups, **3a** and **3b**, and analyzed them by FT-IR, UV-vis and Fluorescence spectroscopy, XRD, and TEM. The optical properties of **3a** and **3b** were measured by UV-vis and fluorescence spectrophotometry. Both **3a** and **3b** showed high solubility in organic solvents such as chloroform, chlorobenzene, and dichloromethane. The quasi-solid DSSC devices prepared with a polymer electrolyte using **3a** or **3b** as an additive showed higher photovoltaic performance than the devices with a polymer electrolyte without **3a** or **3b**, due to their increased J_sc_ values. The best result obtained for the DSSC devices was a power conversion efficiency of 3.62 in the case of the DSSC device using PEG with 3b. Its Voc, J_sc_, and FF were 0.66 V, 10.04 mA/cm^2^ and 0.54, respectively.

## Figures and Tables

**Table 1.** Solubility of TiOPc, **3a**, and **3b.**

Compounds	TiOPc	3a	3b
CHCl_2_	I	S	S
Chlorobenzene	I	S	S
Toluene	I	S	S
Acetone	I	I	I
Methanol	I	I	I
DMF	I	I	I
THF	I	P (17 wt%)	P (26 wt%)

* Solubility in 100 mg/mL

* S = soluble, P = partially soluble, I = insoluble

**Table 2.** XRD data 2 theta angle and relative intensity of **3a** and **3b.**

Compounds	XRD data 2 theta angle (dA)	Relative intensity (%)
	10.94 (8.08)	73
	11.64 (7.59)	79
	17.30 (5.12)	25
	17.92 (4.94)	31
	19.34 (4.58)	84
**3a**	20.46 (4.34)	37
	22.50 (3.95)	28
	22.84 (3.89)	38
	23.50 (3.78)	58
	24.38 (3.64)	100
	26.60 (3.35)	38
	11.32 (7.81)	83
	13.22 (6.69)	41
	16.82 (5.27)	38
	18.04 (4.91)	33
	18.48 (4.80)	96
	19.76 (4.49)	63
**3b**	20.14 (4.41)	77
	21.28 (4.18)	27
	22.06 (4.03)	49
	23.00 (3.87)	79
	23.64 (3.76)	39
	24.86 (3.57)	27
	25.98 (3.43)	100

**Table 3.** The photovoltaic Performances of FTO/TiO_2_/Dye/Electrolyte/Pt Devices Using **3a**, **3a** with PEG, **3b**, and **3b** with PEG under AM 1.5 Illumination.

	V_oc_1) (V)	J_sc_2)(mA/cm^2^)	FF3)	Efficiency (%)
**3a**	0.61	8.49	0.53	2.73
**3a** with PEG4)	0.65	9.84	0.54	3.49
**3b**	0.61	10.02	0.53	3.19
**3b** with PEG4)	0.66	10.04	0.54	3.62
PEG	0.67	8.98	0.49	2.94

1) V_oc_(V): Open circuit voltage.

2) J_sc_(mA/cm^2^): Short circuit current density.

3) FF: Fill factor.

4) Compound : PEG = 1.7 (wt%).
